# Acquired Fanconi Syndrome from Tenofovir Treatment in a Patient with Hepatitis B

**DOI:** 10.1155/2023/6158407

**Published:** 2023-06-17

**Authors:** Shirley X. Jiang, John Duncan, Hin Hin Ko

**Affiliations:** ^1^Department of Medicine, University of British Columbia, Vancouver, Canada; ^2^Division of Nephrology, Faculty of Medicine, University of British Columbia, Vancouver, Canada; ^3^Division of Gastroenterology, Faculty of Medicine, University of British Columbia, Vancouver, Canada

## Abstract

Fanconi syndrome is a rare disease of generalized proximal tubule dysfunction which can be acquired secondary to certain medications, including tenofovir, a commonly used hepatitis B treatment. Signs and symptoms of ensuing renal wasting can be severe but vague, leading to potentially avoidable invasive investigations and delays in diagnosis. We present a case of a 62-year-old female with chronic hepatitis B on tenofovir treatment who was found to have subacute weakness, anorexia, and weight loss. She underwent extensive investigations including computed tomography (CT) imaging, bronchoscopy, upper and lower endoscopy, and psychiatric evaluation. Finally, persistent electrolyte derangements led to urine studies, which demonstrated acquired Fanconi syndrome secondary to tenofovir. After discontinuing tenofovir disoproxil fumarate and starting tenofovir alafenamide, her symptoms resolved and her renal function recovered. This case illustrates the importance of maintaining clinical suspicion for tenofovir-induced Fanconi syndrome, given the common use of tenofovir as first-line hepatitis B treatment and the availability of less nephrotoxic alternatives.

## 1. Introduction

Tenofovir disoproxil fumarate (TDF), a common first-line treatment for hepatitis B, is a rare cause of Fanconi syndrome, a disease of generalized proximal tubule dysfunction. Symptoms include fatigue, weakness, osteomalacia, and weight loss. Laboratory work demonstrates electrolyte abnormalities, a positive anion gap, and unexplained glucosuria or proteinuria. Particular vigilance is required in the presence of older age, low body weight, renal dysfunction, concomitant nephrotoxins, and prolonged treatment duration, though Fanconi syndrome can also develop in the absence of risk factors. Switching from TDF to less nephrotoxic hepatitis B treatments can lead to renal recovery, highlighting the importance of making a timely diagnosis.

## 2. Case Report

A 62-year-old female with hepatitis B presented to the hospital with weakness, anorexia, and 30 pounds of weight loss over the past year. Six years prior, she was started on TDF for active hepatitis B, with complete virological suppression and normal liver enzymes. On admission, laboratory values showed creatinine 1.15 mg/dL, sodium 139 mmol/L, potassium 1.9 mmol/L, phosphate 1.3 mmol/L, and a nonanion gap metabolic acidosis (NAGMA). Urinalysis was positive for glucose, leukocytes, occasional hyaline casts, and trace proteins.

She had extensive investigations, with an unremarkable CT neck, thorax, abdomen and pelvis, normal bronchoscopy, esophagogastroduodenoscopy, and colonoscopy. Her hepatitis B virus (HBV) level was undetectable, and alpha-fetoprotein, autoimmune panel, and C-reactive protein were all within the normal ranges. The psychiatry team was consulted to ensure she had no signs of disordered eating.

After more than one week of hospitalization, she continued to have hypokalemia and hypophosphatemia, which were initially attributed to refeeding syndrome. Upon further investigation, urine electrolytes demonstrated sodium 99 mmol/L, potassium 48.4 mmol/L, and chloride 124 mmol/L, with a urine anion gap (UAG) of 23.4 mmol/L. She also had an elevated urine phosphate-to-creatinine ratio, urine urate-to-creatinine ratio, and urine protein-to-creatinine ratio, corresponding with proteinuria >1 g/day though urinalysis still demonstrated trace protein. The positive UAG, serum NAGMA, glucosuria, phosphaturia, uricosuria, and proteinuria led to a diagnosis of acquired Fanconi syndrome due to TDF.

TDF was discontinued, and she was discharged home on electrolyte replacements. Three months following her admission, for her chronic hepatitis B infection, she was started on tenofovir alafenamide (TAF), a different tenofovir prodrug that is not associated with Fanconi syndrome. Five months later, her electrolyte levels normalized, urine studies were negative, and she gained 15 pounds with an improvement in her energy level. Now, more than a year since the discontinuation of TDF and start of TAF, her renal function remains stable and her hepatitis B viral load remains undetectable.

## 3. Discussion

Fanconi syndrome is characterized by global dysfunction of the proximal tubule, leading to renal wasting of electrolytes, bicarbonate, glucose, uric acid, and amino acids. Adult-onset presentations are typically acquired due to monoclonal gammopathies, renal transplantation, and medications such as tenofovir, a first-line treatment for hepatitis B [1]. Symptoms correspond to deficiencies from renal losses and can include fatigue, weakness or myalgias, osteomalacia, fractures, and weight loss. To establish a diagnosis, urine studies demonstrate renal wasting of phosphate, uric acid, protein, glucosuria, and bicarbonate, which is demonstrated by a positive UAG or low urine ammonia. Importantly, urinalysis is insufficient to detect tubular protein loss; as seen in our case, the patient only had trace protein on urinalysis but had >1 g/day on the urine protein-to-creatinine ratio [2]. Management of Fanconi syndrome is comprised of electrolyte replenishment and addressing underlying causes. Serum electrolytes, bicarbonate, and urine studies should be followed monthly to ensure adequate replacement and monitor for recovery. Vitamin D and B12 levels should be checked and supplemented if needed [3].

TDF, a nucleotide reverse transcriptase inhibitor, is a rare but well-established cause of acquired Fanconi syndrome. The mechanism is thought to be interference by TDF with tubular transport, leading to drug accumulation and mitochondrial toxicity in the proximal tubule. Risk factors for tenofovir-associated nephrotoxicity are increased age, low body weight, pre-existing renal dysfunction, concomitant use of nephrotoxic drugs, prolonged treatment duration, and polymorphisms in tubular transporter genes [4, 5]. However, Fanconi syndrome can develop in the absence of risk factors and at any time during therapy, such as in our patient. Other drugs associated with the development of Fanconi syndrome include adefovir, ranitidine, mercaptopurine, cisplatin/carboplatin, isofosphamide, aminoglycoside and tetracycline antibiotics, and valproic acid [3].

The European Association for the Study of the Liver (EASL) and Infectious Diseases Society of America (IDSA) recommend vigilance during tenofovir treatment by ensuring nephrotoxin avoidance, renal dosing, and close monitoring of renal function. Urine studies should also be monitored if there is concomitant chronic kidney disease or multiple antivirals used. Serum creatinine and electrolytes, including phosphate, should be obtained every 3 months in the first year and every 6 months thereafter [6, 7]. TDF should be discontinued for serum phosphate below 1 mg/dL or creatinine clearance below 50 mL/min [6]. Stopping TDF typically leads to renal recovery over months, though there have been reports of persistent renal impairment [8].

Most reports of tenofovir-induced Fanconi syndrome are secondary to TDF, and renal recovery has been observed after switching to another tenofovir prodrug, tenofovir alafenamide (TAF) [9]. This is attributed to TAF having higher potency despite 90% lower plasma concentrations and not being a substrate for tubular transporters, thus preventing accumulation [10]. To our knowledge, there has been a single case report suggesting that TAF may also be associated with Fanconi syndrome but the renal history of this patient is unclear [11]. Currently, TAF-associated nephrotoxicity is considered very rare compared to TDF.

Despite vague symptoms and a lack of risk factors for tenofovir toxicity, this case highlights the importance of maintaining clinical suspicion for tenofovir-associated Fanconi syndrome to make a timely diagnosis, especially given the availability of less nephrotoxic alternatives in the treatment of hepatitis B.

## Data Availability

All relevant patient data pertaining to the case are included in the article and there were no additional sources of data.
